# Viral dissemination and diagnostic insights from experimental infection of sheep with Crimean-Congo hemorrhagic fever virus

**DOI:** 10.1038/s44298-026-00228-y

**Published:** 2026-08-24

**Authors:** Benjamin Gutjahr, L. Mathias Michaely, Caroline Bost, Balal Sadeghi, Filipe Tomaz, Markus Keller, Sven Reiche, Ali Mirazimi, Jürgen A. Richt, Lisa Hensley, Sandra Diederich, Mathias Streitz, Anne Balkema-Buschmann, Martin H. Groschup, Kerstin Fischer

**Affiliations:** 1 Institute of Novel and Emerging Infectious Diseases, Friedrich-Loeffler-Institut, Federal Research Institute for Animal Health, Greifswald-Insel Riems, Germany; 2 Department of Experimental Animal Facilities and Biorisk Management, Friedrich-Loeffler-Institut, Federal Research Institute for Animal Health, Greifswald-Insel Riems, Germany; 3 Department of Medicine, Karolinska Institutet, Stockholm, Sweden; 4 Diagnostic Medicine/Pathobiology, College of Veterinary Medicine, Kansas State University, Manhattan, KS USA; 5 Department of Veterinary Tropical Diseases, Faculty of Veterinary Science, Onderstepoort, University of Pretoria, Pretoria, South Africa; 6 Zoonotic and Emerging Disease Research Unit, National Bio and Agro-Defense Facility, USDA Agricultural Research Service (ARS), Manhattan, KS USA

**Keywords:** Diseases, Immunology, Microbiology

## Abstract

Crimean-Congo hemorrhagic fever virus (CCHFV) is a tick-borne orthonairovirus widely distributed across Asia, Africa, the Middle East, and Southern Europe, causing severe illness and hemorrhagic fever in humans. While sheep usually show no clinical symptoms, they develop viremia and can play a key role in transmitting the virus to ticks and humans. Despite their importance in transmission, our understanding of infection dynamics in these animals remains limited. To bridge this knowledge gap, we conducted a controlled study in which six Pomeranian Coarsewool sheep were experimentally infected with CCHFV, strain Kosovo Hoti, and monitored over 21 days. All sheep developed transient fever and viremia. Necropsies at 5–7 days post infection (dpi) revealed highest viral loads in liver, spleen, testicles, and lymphoid tissues. Seroconversion occurred by 7 dpi and virus-neutralizing antibodies were detectable by day 8. These findings enhance our understanding of CCHFV infection dynamics in sheep and will aid future research, including vaccine development.

## Introduction

Crimean-Congo hemorrhagic fever virus (CCHFV) is a tick-borne, zoonotic orthonairovirus belonging to the family *Nairoviridae* within the class of *Bunyaviricetes*. CCHFV is the causative agent of Crimean-Congo hemorrhagic fever (CCHF), a febrile illness in humans that can progress to a severe viral hemorrhagic fever with mortality rates up to 30%^1^. Until today, there are no widely approved vaccines or targeted antiviral therapies available^1,2^. The geographic distribution of the virus is widespread and strongly depends on the presence of its tick vector and reservoir, namely ticks of the *Hyalomma* genus^1^. Of note, global warming and the movement of migratory birds are widely thought to contribute to the geographical expansion of these ticks into regions that were previously negative, thereby increasing the risk of CCHFV infections beyond historical borders. For example, the first autochthonous human case in Spain was reported to have occurred in 2013^3^. Since then, molecular and serological evidence have strongly suggested endemic circulation of the virus among certain tick species and wildlife in several areas of Spain^4–7^. Moreover, the first and fatal human case in Portugal, the recent serological detection of CCHFV in cattle and wildlife in France, and the first molecular detection of CCHFV in *Hyalomma* ticks in Southern France further underline the spread and increasing impact of the virus on public and veterinary health in Southern Europe^8,9^.

Human exposure to the virus is reported to occur primarily via the bite of an infected tick. However, contact with viremic blood or tissues from infected animals can also lead to infection^1,10–12^. While the severe clinical disease is exclusive to humans, subclinical CCHFV infection in animals, including sheep and other ruminants, is thought to play an important role in the epidemiology of the virus^13–15^. These animals typically remain asymptomatic but can develop viremia and thus serve as amplifying hosts, contributing to the transmission of the virus to both ticks and humans^1,10,11,16^. Due to effective seroconversion, they are often used as sentinels for serosurveillance studies^17^. Despite the growing importance of CCHFV as a tick-borne zoonosis, comprehensive studies on the course of infection in ruminants remain limited.

Field reports on CCHFV in ruminants are often based on molecular and seroepidemiological studies, in which typically only infested ticks and ruminant blood samples are collected and analyzed. These studies usually aim to detect the presence and geographical distribution of the virus, or to assess the prevalence of virus-specific antibodies, rather than examining the animals themselves for potential clinical symptoms^18–21^. Moreover, viremic animals in the field are often only detected or suspected retrospectively, for example, when a person falls ill and the illness is linked to potential exposure to infected animals or virus-contaminated slaughter material^22–24^. In addition, drawing conclusions about seroconversion or antibody kinetics from these studies can still be challenging, especially if the exact timing and frequency of virus exposure remain unknown.

Published experimental studies on CCHFV infection in farm animals that address these gaps are scarce, with most research dating back to the 1990s or earlier^15,25–27^. The conclusions from these studies are also limited by inconsistencies in the viral strains used, many of which were poorly characterized or of uncertain pathogenicity^15^. In addition, most of these early results have yet to be verified using modern laboratory techniques. A recent study by Li et al. investigating host responses and virus dissemination in experimentally infected sheep^28^, demonstrates renewed efforts in this field. Nevertheless, such investigations remain limited overall, largely due to the classification of CCHFV as a BSL-4 pathogen and the limited number of high-containment laboratories capable of conducting these experiments. However, a better understanding of the detailed course of CCHFV infection in sheep, including viral replication, tissue tropism, and antibody kinetics is essential for improving diagnostic methods and developing preventive and therapeutic interventions.

In the current study, we experimentally infected six sheep of a local German breed (Pomeranian Coarsewool sheep) with a clinical CCHFV strain isolated from a fatal human case in Southeastern Europe (strain Kosovo Hoti). We provide detailed insights into the time course of CCHFV infection in sheep, with a particular focus on viral dissemination in the host and the humoral immune response, which supports the development of better and more targeted serological diagnostics for future use in the field.

## Results

### Local German sheep develop short-term viremia but no significant virus shedding after inoculation with CCHFV

**Fig. 1:**
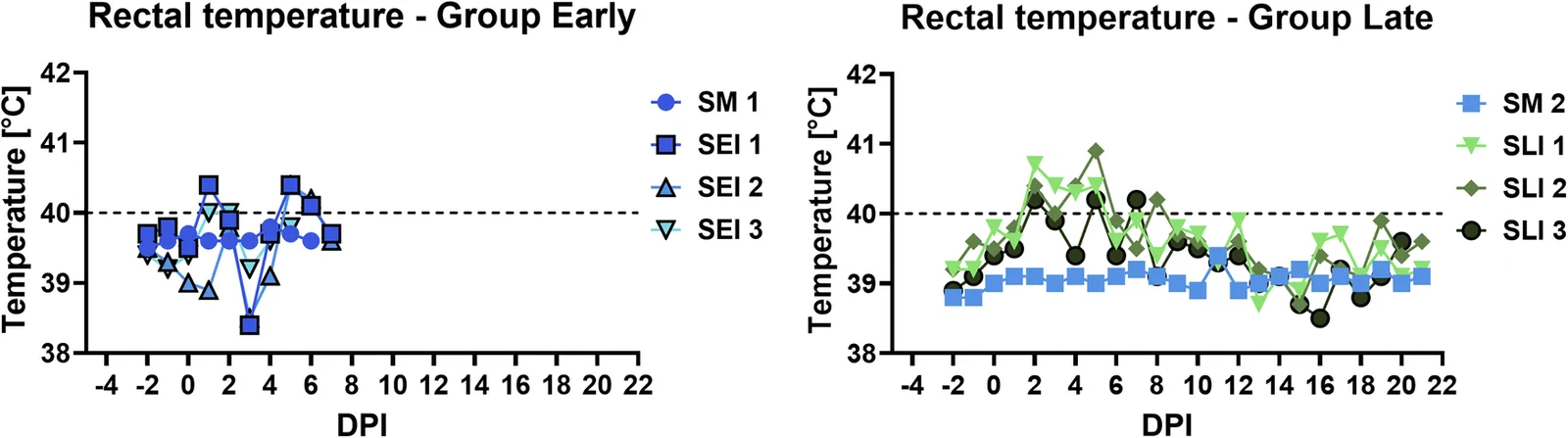
Overview of the rectal body temperature. The rectal body temperature of each sheep from the early and late group was recorded daily.

**Fig. 2:**
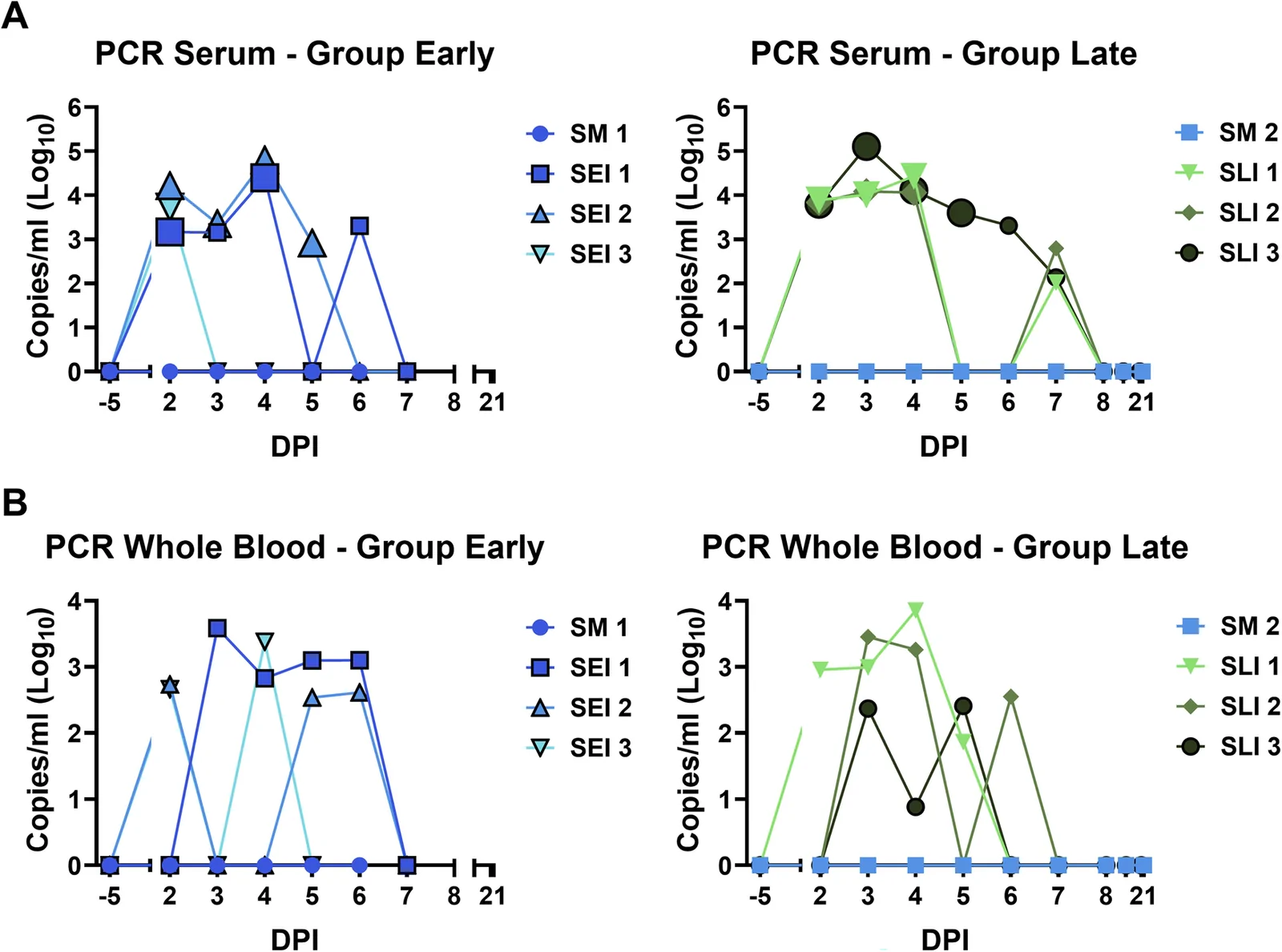
Overview of measured viremia. CCHFV RNA loads determined by RT-qPCR in serum (**A**) and whole blood (**B**) from sheep of the early and late group. Copy numbers are expressed as log₁₀ copies per milliliter (ml) serum/whole blood. Enlarged symbols represent samples, which showed viral replication in virus titration.

Six five-month-old sheep were inoculated with 2 × 10^5^ TCID_50_ CCHFV Kosovo Hoti via a combined subcutaneous and intravenous route (Supplementary Fig. 1). All six sheep exhibited a brief period of elevated body temperature during the first week, with the earliest onset at 1 day post infection (dpi) (Fig. 1), accompanied by mild loss of appetite and slightly labored breathing (Supplementary Table 1). During this early phase, between 2 and 7 dpi, all sheep developed a transient viremia with a peak between 3 and 4 dpi (Fig. 2). Viral RNA levels were generally higher in serum than in whole blood, with maximum concentrations reaching approximately 127,000 RNA copies/ml in serum and about 7000 RNA copies/ml in whole blood (Fig. 2A, B). Virus titration of serum samples collected up to 5 dpi confirmed the presence of circulating, infectious virus with titers reaching up to 10^4^ TCID_50_/ml (Supplementary Table 2). Notably, virus was successfully isolated even from serum samples containing approximately 800 RNA copies/ml. An overview of the virus titration can be seen in Supplementary Table 2.

A viral isolate was obtained from the serum of sheep SLI 3, collected at 3 dpi, which showed the highest viral burden by RT-qPCR. Following a single passage in cell culture, the isolate was subjected to deep sequencing. Notably, the virus stock used for inoculation harbored ten synonymous mutations relative to the reference genome sequence (Supplementary Table 3). However, only one of these mutations, located in the L segment (nt 3148 A > G), was retained in the sheep-derived isolate. Additionally, two novel synonymous mutations were identified in the SLI 3 sheep isolate: one in the M segment (nt 3445 G > A) and one in the L segment (nt 2964 C > T), both absent from the original inoculum (Supplementary Table 4). The remaining nine synonymous mutations present in the inoculum were no longer detectable in the isolate recovered from the sheep.

Shedding of viral RNA was detected only in two nasal swabs derived from different animals, between 4 and 5 dpi (Supplementary Fig. 2A–C). Despite high RNA copy numbers of approximately 10,000 and 50,000 RNA copies/ml in the nasal swab samples, no infectious virus was recovered from this material.

Serum chemistry analysis in one mock and three CCHFV-infected animals, monitored throughout the study up to 21 dpi, revealed no obvious evidence of liver or kidney dysfunction. Minor changes were observed for both mock and infected animals, suggesting these were not virus-related (Supplementary Table 5).

### Viral RNA present across multiple tissues but only temporarily

**Fig. 3:**
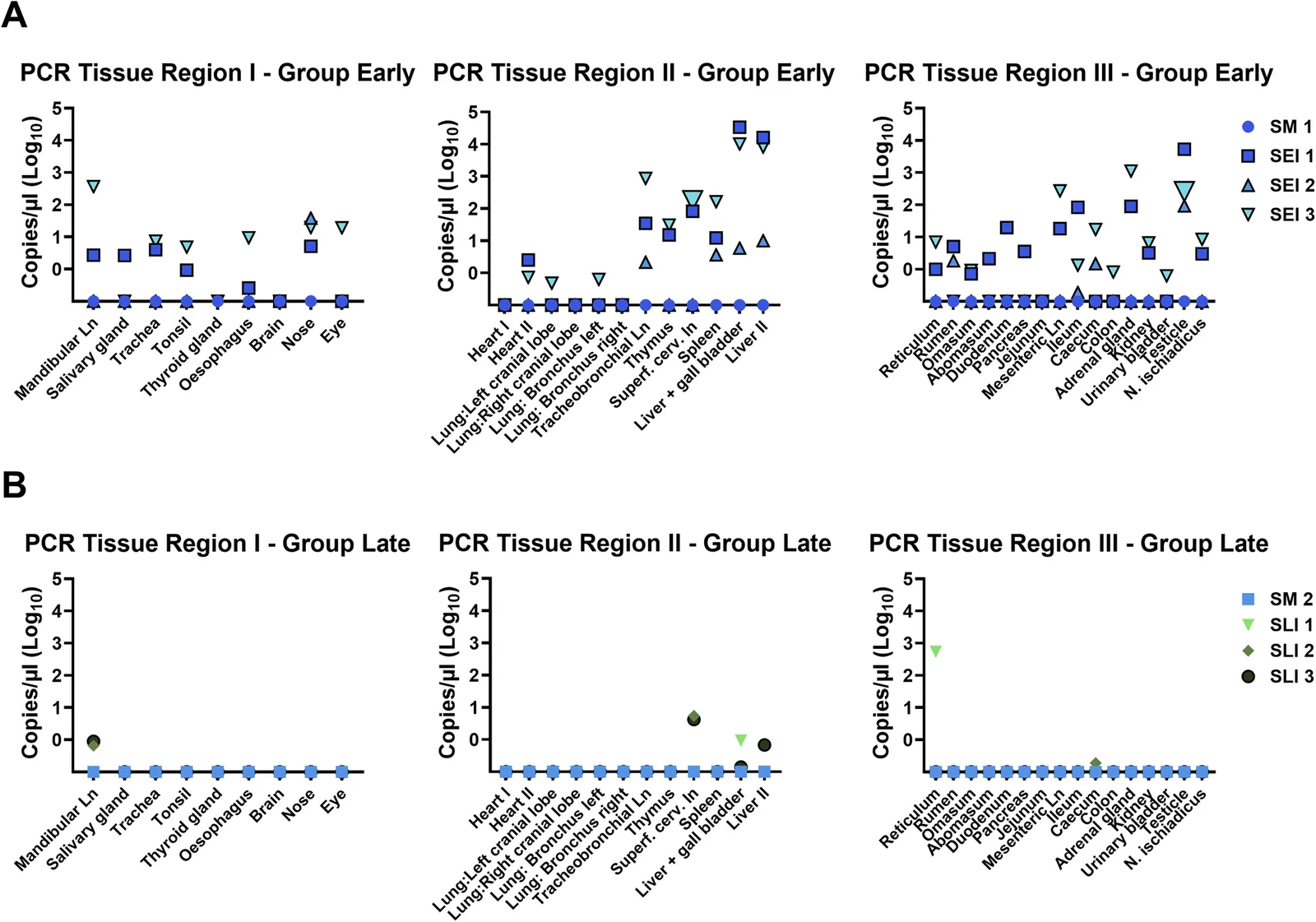
Overview of detected viral loads in tissue samples. CCHFV RNA loads determined by RT-qPCR in tissue samples collected at early (**A**) and late (**B**) timepoints of necropsies. Copy numbers are expressed as log₁₀ copies per microliter (µl) of RNA. Enlarged symbols represent samples, which showed viral replication in virus titration.

To examine virus tissue distribution over the course of infection, three animals were necropsied at an early stage, during or shortly after the viremic stage (between 5 and 7 dpi), while the remaining three animals were euthanized and sampled at 20 and 21 dpi. No gross pathological lesions were observed in major organs collected during necropsy. Viral loads in tissue samples collected at five and seven dpi were notably higher than those collected at 20 and 21 dpi (Fig. 3A, B). The highest viral loads were detected in the liver, spleen, testicles, and various lymph nodes sampled at the necropsies performed at early timepoints (Fig. 3A). Virus titration revealed the presence of replication-competent virus in samples from the testicle and cervical lymph node collected at 5 dpi (SEI 3). Surprisingly, despite high viral loads in the liver samples collected during necropsies at early timepoints, infectious virus could not be recovered from these samples. By 20 and 21 dpi, viral RNA had been largely cleared from the organs, with only residual amounts of viral RNA detected in the liver and cervical lymph node (Fig. 3B).

**Fig. 4:**
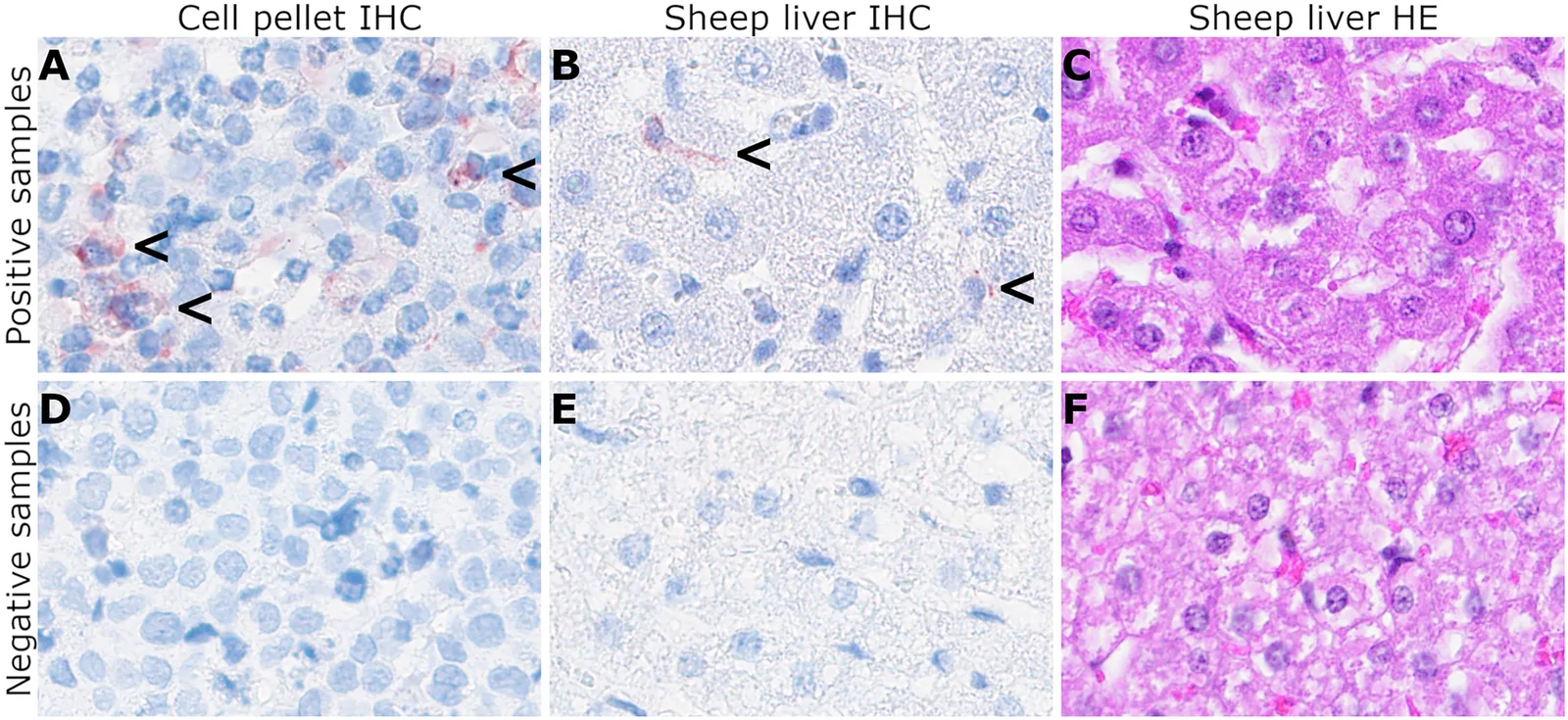
Histopathological findings. Histopathology reveals viral antigen in a CCHFV infected SW-13 cell pellet marked by arrowheads (**A**). Likewise, viral antigen is detected in the liver of sheep SLI 1 (**B**), but no inflammatory or necrotizing lesions are detected in the HE staining (**C**). Negative cell pellet (**D**) and a mock infected sheep (SM 1, **E,** **F**) are shown in the bottom row. CCHFV. Crimean-Congo hemorrhagic fever virus; IHC: Immunohistochemistry; HE: Hematoxylin-and eosin staining. Magnification, 40x.

Histopathological examination of formalin-fixed organ sections revealed minimal to mild lympho-histiocytic interstitial pneumonia (7/8 animals), tapeworm infection and eosinophilia in the intestinal samples (6/8 animals). Further findings included lymphohistiocytic and eosinophilic infiltrates of minimal to mild degree in the liver (4/8 animals), kidney (2/8 animals) and adrenal gland (1/8 animals) as well as non-reactive sarcosporidia in the myocardium of 1/8 animals. The skin samples at the site of injection exhibited minimal to moderate inflammatory cell infiltrations of a mixed-cellular character with histiocytic dominance in most animals. The detailed histopathology scoring is provided in Supplementary Table 6. Immunohistochemical (IHC) staining for CCHFV N and Gc antigen was present in liver and testis of sheep SLI 1 (Fig. 4) and liver, spleen and testis of sheep SLI 3. No IHC staining was detected in the tissues of the other animals.

### Immune response to CCHFV infection

**Fig. 5:**
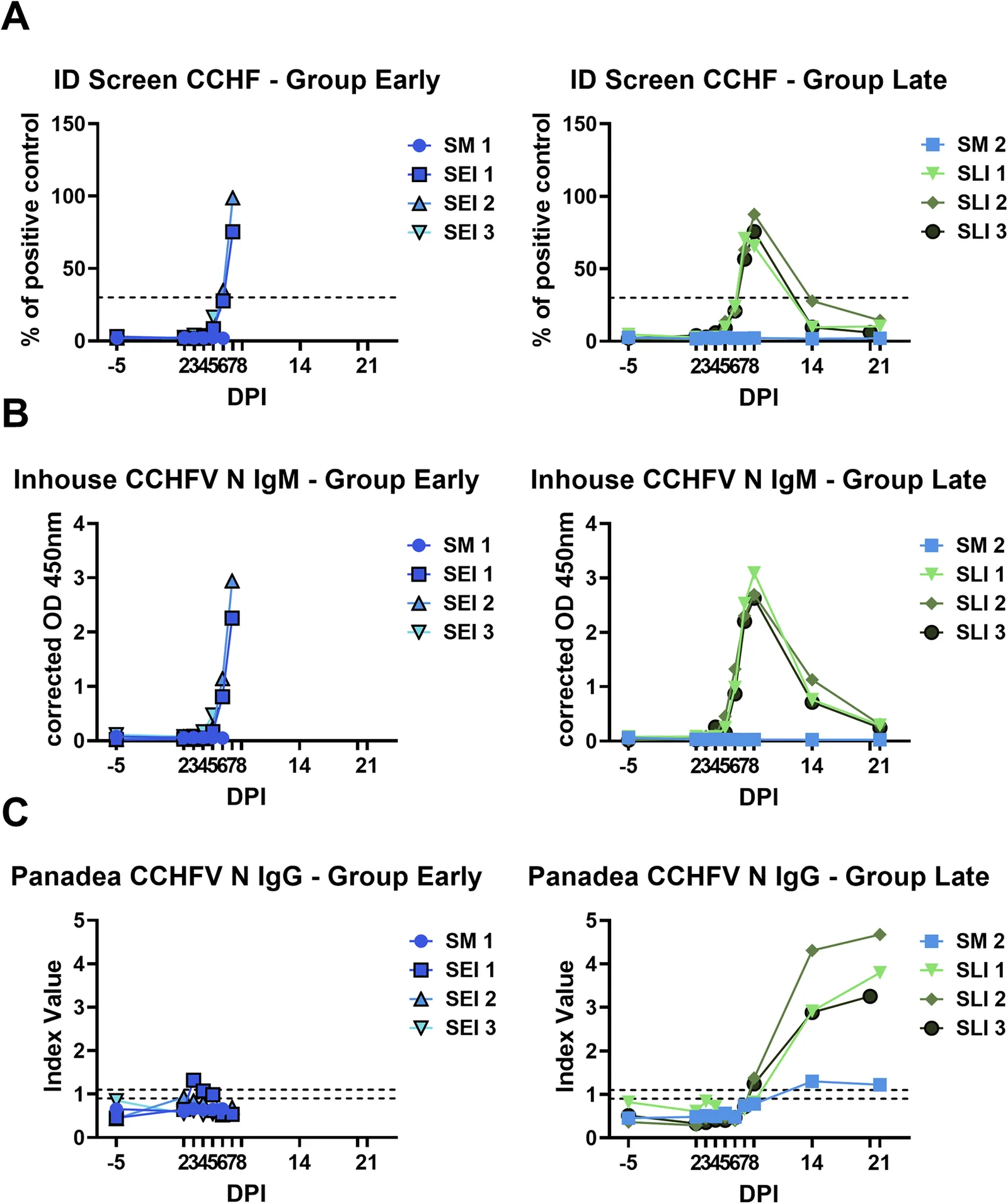
Overview of serological assays targeting CCHFV N. Detection of CCHFV N - specific IgG and/or IgM antibodies of sheep from early and late group using commercial and in-house tests (**A**–**C**). The commercial ID Screen® CCHF ELISA was used to detect both IgG and IgM (**A**). The in-house ELISA was developed and performed for the detection of anti-N-specific IgM (**B**). The commercial Panadea CCHFV IgG ELISA kit was used to specifically measure IgG (**C**). Dotted lines represent the cutoff according to the manufacturer´s instructions.

**Fig. 6:**
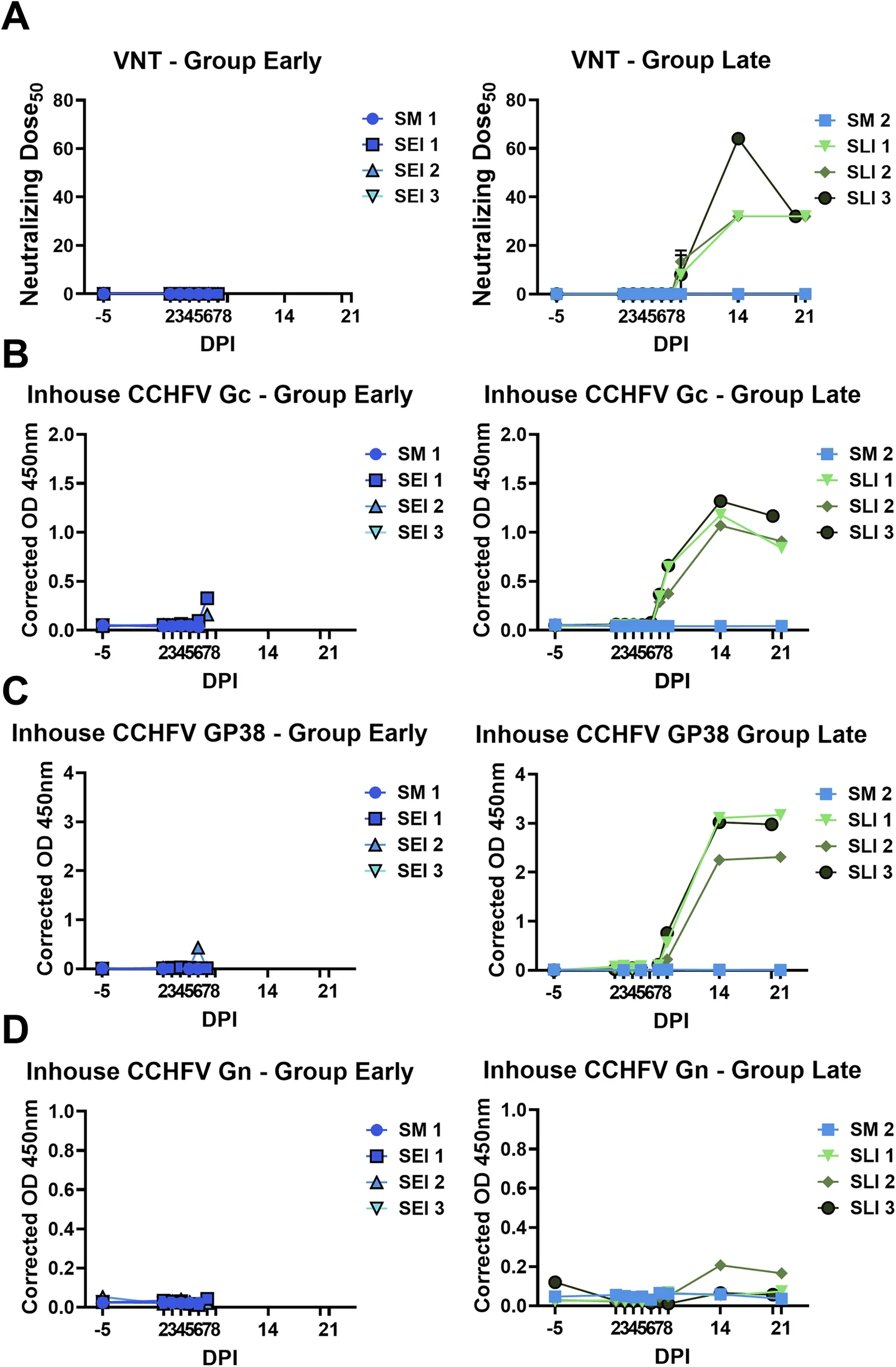
Overview of serological assays targeting antigens of CCHFV GPC. Detection of CCHFV glycoprotein-specific IgG antibodies of sheep from early and late group using in-house tests (**A**–**D**). Virus-neutralizing antibody titers were determined in virus neutralization test (VNT; **A**). In-house indirect ELISAs were performed to detect specific antibodies against Gc (**B**), GP38 (**C**) or Gn (**D**).

We investigated the CCHFV-specific antibody response using commercially available and in-house CCHFV-specific ELISAs, along with a virus neutralization test (VNT). Depending on the antigen used, seroconversion to the viral nucleoprotein (N) and glycoproteins started between 7 and 8 dpi (Figs. 5 and 6). Sheep sera were first tested for N-specific antibodies using the ID Screen® CCHF Double Antigen ELISA, which has been widely used in various field studies across numerous species due to its species-independent testing principle and its design to detect both IgM and IgG, according to the manufacturer^29,30^. Interestingly, after detecting the first N-specific antibodies around 7 and 8 dpi, antibody titers already dropped below the detection limit by 14 dpi (Fig. 5A). Since the commercial test does not distinguish between IgM and IgG antibodies, we developed an in-house anti-N IgM ELISA and additionally screened the sheep sera using this test. Intriguingly, the antibody kinetics observed with the in-house IgM-specific assay were highly similar to those detected by the ID Screen® ELISA (Fig. 5B), suggesting that, in this experimental setting, the ID Screen signal may have been largely driven by early IgM responses. To confirm that the CCHFV-infected sheep had developed a virus-specific IgG response, we used an N-specific commercial ELISA (Panadea Diagnostics GmbH) that, according to its test principle, exclusively detects CCHFV-specific IgG antibodies^31^. Here, only sheep serum samples collected at later time points, i.e., 14 and 21 dpi, were positive and thus confirmed the presence of CCHFV-specific IgG antibodies in the serum samples (Fig. 5C).

The virus-neutralizing activity in the serum samples was analyzed by VNT, while the specific IgG response to individual glycoproteins, i.e., against GP38, Gn and Gc, was assessed by indirect in-house ELISAs. Serum samples showed virus-neutralizing activity starting at 8 dpi (Fig. 6A). Titers peaked around day 14, though overall titers remained relatively low. Only one serum sample from a single animal reached a maximum titer of 1:64, while all the other sera showed titers of 1:32 or lower (Fig. 6A). Anti-Gc antibodies were first detected around 7 dpi, they increased over time, and showed a slight decline at 21 dpi (Fig. 6B). A clear antibody response against GP38 was detected starting around 8 dpi, and reached a robust plateau by 14 dpi (Fig. 6C). While both GP38 and Gc were shown to elicit a strong humoral response in the sheep, no clear reactivity against Gn was detected (Fig. 6D).

**Fig. 7:**
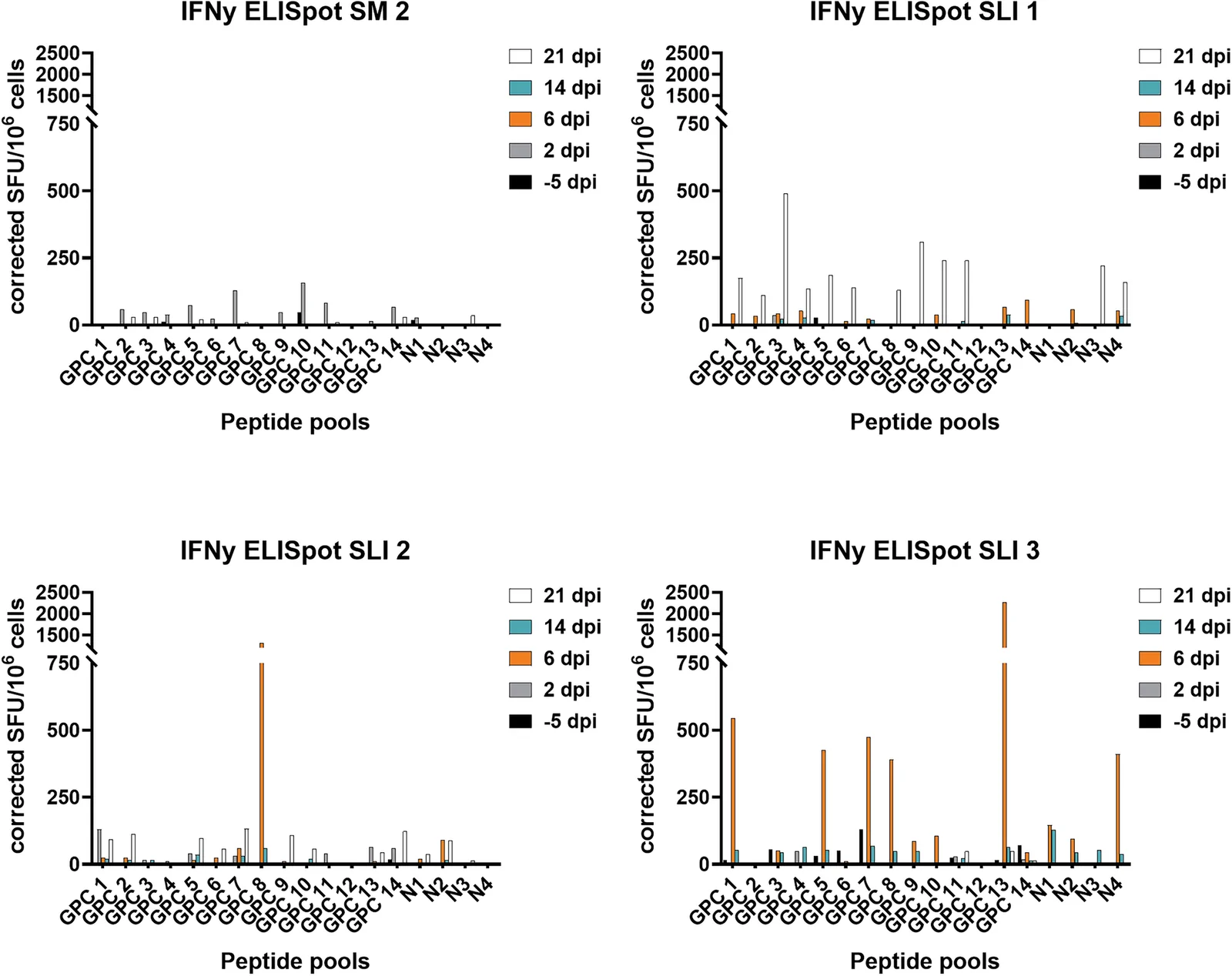
Overview of cellular response against CCHFV. PBMCs from mock- (SM 2) and CCHFV-infected sheep (SLI 1-3) were collected at indicated days post infection (dpi). CCHFV-specific T-cell responses were measured by IFN-γ ELISpot using peptide pools spanning CCHFV GPC and N. PBMCs per animal and DPI were analyzed in duplicate. Data show the mean corrected peptide-pool specific spot forming units (SFUs), normalized to 10^6^ cells, and calculated by subtracting background SFUs from unstimulated PBMC wells.

To investigate the kinetics of CCHFV-specific T cell responses in the sheep, interferon-gamma (IFN-γ)-secreting cells among peripheral blood mononuclear cells (PBMCs) were quantified using ELISpot assays. PBMCs collected at multiple time points post-infection from three CCHFV-infected sheep and one mock-infected control (SM 2, SLI 1–3) were stimulated in vitro with peptide pools spanning the CCHFV GPC and N, and the resulting IFN-γ responses were measured. Stimulation of PBMCs with CCHFV-specific peptide pools resulted in detectable IFN-γ responses in all three infected sheep, confirming the induction of antigen-specific T cell responses (Fig. 7). Overall, the reactivity pattern varied between individuals, and neither a specific dpi nor a specific peptide pool consistently elicited a dominant response across all animals, indicating a heterogeneous and individual T cell recognition profile in the sheep. While low-level IFN-γ secretion was also observed in the mock-infected control animal, the magnitude of responses in CCHFV-infected animals was markedly higher, supporting the specificity of the antigen-induced T cell responses.

## Discussion

Natural CCHFV infection in sheep has been reported to be asymptomatic and thus typically goes unnoticed under field conditions^1^. However, infected ruminants have been identified to play a substantial role in transmitting CCHFV to ticks and humans, with the latter developing severe and potentially fatal disease^10,11,16^. Therefore, gaining a deeper insight into CCHFV infection dynamics in sheep is essential for a better understanding of the virus epidemiology and its interaction with susceptible hosts.

CCHFV is naturally transmitted to livestock through tick bites. However, recapitulating the natural route of infection under experimental conditions is rather difficult, as maintaining a viable tick colony within BSL4 containment and achieving a reliable infection rate among sheep using experimentally infected ticks present significant challenges. Therefore, a combined subcutaneous and intravenous inoculation route was selected as a reproducible experimental approach to ensure productive infection and comparability with previous studies. This inoculation strategy using the clinical CCHFV isolate Kosovo Hoti had previously been used successfully in non-human primates (NHPs), such as cynomolgus and rhesus macaques^32,33^, and more recently in sheep, leading to productive infection^28^. Given these results, the Kosovo Hoti strain and the combined inoculation route were selected for the present study.

Possible positive effects of tick saliva, which could not be included in any of these experimental animal studies, are believed to play a role in the course of CCHFV infection and to date have only been addressed in a single previous in vitro study^34^. For other tick-borne viruses, such as some neurotropic orthoflaviviruses or severe fever with thrombocytopenia syndrome virus (SFTSV), tick saliva has been shown to modulate and promote viral replication in different cell types, and to enhance virus transmission in experimentally infected mice^35–38^. Therefore, it remains to be explored whether tick saliva has enhancing effects on CCHFV replication or transmission in sheep. Nonetheless, the absence of tick saliva and the use of an artificial inoculation route must be considered when interpreting the data of this study. Additionally, this study involved a single inoculation under controlled conditions, whereas in the field, multiple ticks may infest a host simultaneously or within short intervals, potentially resulting in several bites from infected ticks and a booster effect on the infection.

Seroconversion in this study was marked by the appearance of IgM antibodies directed against the viral N as early as 7 dpi. By approximately 8 dpi, both glycoprotein- and N-specific IgG antibodies were detected. Interestingly, the antibody kinetics observed with the ID Screen® test, which is designed to detect both IgM and IgG anti-N antibodies, were highly similar to those detected by the in-house IgM ELISA, suggesting that, in the present experimental setting, the ID Screen® signal may have been largely driven by early IgM responses. These observations are particularly notable, considering the widespread use of the ID Screen® test in serosurveillance studies. Interestingly, serological studies using this test often report lower anti-CCHFV antibody seroprevalence rates in sheep compared to cattle^20,39–41^. Notably, a similar observation was reported in a bluetongue virus (BTV) study involving infected ruminants and alpacas, in which the signal of two commercial double-antigen ELISAs declined markedly shortly after the IgM peak. The authors suggested that this phenomenon may be associated with the transition from IgM to IgG antibodies, although the underlying mechanism remains unclear and requires further investigation^42^. In contrast, the other commercial assay (Panadea Diagnostics GmbH), designed to detect only IgG, showed a clear anti-N IgG antibody response from approximately 8 dpi onward. Together, these findings indicate that different serological assays may detect distinct phases or components of the antibody response in sheep, which could influence apparent seropositivity in surveillance studies.

In the current study, no clear anti-Gn-antibody response was detected in the serum of the infected sheep using the in-house ELISA. This contrasts with data from a recent study by Li et al. in which sheep experimentally infected with CCHFV developed a strong anti-Gn antibody response detected by indirect ELISA^28^. However, our results align more with observations from other CCHFV studies, which show reduced antibody responses against CCHFV Gn in humans, ruminants, and mice compared to other antigens^43–45^. Since the in-house ELISA used a commercially obtained Gn antigen derived from the heterologous IbAr 10200 strain rather than the Hoti strain used for infection, strain-specific antigenic differences may have contributed to the limited Gn reactivity observed here. However, in sheep infected with the closely related orthonairovirus Nairobi sheep disease virus (NSDV), anti-Gn antibodies were detected only in a subset of the sheep, while all infected sheep developed a robust neutralizing as well as a strong anti-GP38 antibody response^46,47^. In contrast to Gn, antibodies against CCHFV Gc, the primary target of virus-neutralizing antibodies, were detected with the in-house ELISA using a commercially obtained IbAr 10200-derived antigen. Despite observing neutralizing activity of the sheep sera by VNT, the overall neutralizing titers were comparatively low and already appeared to decline towards the end of the study, which may suggest their limited role in controlling the infection. In contrast, the recent study by Li et al. on experimental infection of sheep with CCHFV reported considerably higher neutralizing titers^28^. However, several factors complicate a direct comparison of the two studies and their respective findings, including differences in the VNT setup, the breed and age of the sheep, and a tenfold higher virus dose used for inoculation, which may contribute to the observed differences.

In contrast to the report by Li et al. (2024), the sheep in our study exhibited only residual RNA levels in tissues collected at later necropsy time points, with no evidence of substantial viral RNA persistence across various tissues up to 20–21 dpi in this experimental setting. Moreover, the present study only detected low amounts of viral antigen by immunohistochemistry further supporting a transient and self-limiting infection course. These findings differ from the broader tissue dissemination and longer viral RNA persistence reported previously, despite the use of the same CCHFV Kosovo Hoti strain^28^. The divergent findings between the studies may reflect differences in host-associated factors, including sheep age and breed, as Li et al. used 2-month-old sheep whereas the present study used 5-month-old animals. These differences may have influenced the course of infection due to age-related variations in immune responses. In addition, differences in experimental design, including sampling schedules, methodological approaches, and the approximately 10-fold higher inoculation dose used by Li et al. may also have contributed to the observed discrepancies. Collectively, these observations highlight the variability of experimental CCHFV infection in sheep and the importance of comparative studies using standardized approaches.

The isolated PBMCs of the sheep in this study exhibited variable and generally modest IFN-γ responses upon stimulation with viral peptide pools, suggesting a heterogeneous T cell activation profile among individuals. This variability may be influenced by genetic diversity in the ovine major histocompatibility complex (MHC), known as the Ovine Leukocyte Antigen (OLA) system, which encompasses highly polymorphic class I and class II genes responsible for antigen presentation^48^. The OLA polymorphism in outbred sheep populations is likely to contribute to individual differences in T cell epitope recognition and immune responsiveness. Additionally, as intermediate hosts, sheep develop transient, short-lived viremia without clinical signs and may thus rely on innate immunity or humoral responses to control infection. In contrast, splenocytes from immunocompetent mice infected with a mouse-adapted CCHFV strain have shown strong IFN-γ responses, particularly against specific peptide pools within the GPC, including regions in the nonstructural protein (NSm) and the N-terminus of the Gc protein. These responses peaked around 8 dpi and persist up to 28 dpi^49^. The overall differences observed in the immune profiles between sheep and mice indicate differences in host-pathogen interactions and suggest that while adaptive T cell immunity has been shown to be critical in murine models, it may play a less prominent role in ruminant hosts.

The highest viral loads were found in the serum, as well as in liver and spleen, which are also the primary target organs in NHPs and mice, both commonly used as animal models of CCHF disease in humans^32,49,50^. Liver involvement is considered a key contributor to CCHFV pathogenesis, as hepatic dysfunction can impair the synthesis of coagulation factors, and thus promote coagulopathy^1^. However, in contrast to NHPs, mice or clinical CCHFV cases, the CCHFV-infected sheep in this study showed no inflammatory or necrotizing liver alterations and only minimal viral antigen detection by immunohistochemistry in the organ samples of sheep SLI 1 and SLI 3, despite significant viral RNA load in the liver. In addition, we observed mild histologic lesions in the gastrointestinal tract due to parasite infection and multifocal pneumonia in some sheep. Based on the hepatocentric pathology expected from clinically apparent CCHF in rodent and macaque models^1^, these findings are likely to be background lesions within the flock of sheep as illustrated by the mock-infected control animals, which also exhibited these changes. Although an influence of the parasite and lung infection on the immune status, susceptibility to infection, and immune response cannot be completely ruled out, these are most likely conditions often experienced in the field. Similarly, as reported from the field, the sheep exhibited only very mild clinical symptoms, which would unequivocally go unnoticed under field conditions.

Viral RNA was predominantly detected in the serum and in organ samples collected at early time points post-infection. Surprisingly, despite high viral loads in sheep liver samples reaching up to approximately 33,000 RNA copies per µl of extracted RNA, no infectious virus was isolated from these tissues. In contrast, infectious virus could be isolated from serum samples with viral RNA copy numbers as low as <1 RNA copy/µl, suggesting that infectious CCHFV can be efficiently recovered from serum even at very low RNA levels. Interestingly, this finding aligns with another recent report on the experimental CCHFV infection in sheep, which showed that viral RNA levels in tissue samples did not always correlate with the presence of infectious virus^28^. However, technical and tissue-specific factors, including cytotoxicity, inhibitory substances, freeze–thaw effects, or differences in sample processing, may also have influenced virus recovery from tissue homogenates. Therefore, negative virus isolation results should be interpreted cautiously and do not necessarily indicate the complete absence of infectious virus in the respective tissues. Furthermore, direct quantitative comparisons of viral RNA load between different organ types should be interpreted cautiously due to tissue-specific differences in sample processing and RNA recovery. In addition, the limited animal numbers, the controlled experimental infection setting, and methodological constraints associated with high-containment studies represent important considerations when interpreting the findings of the present study.

The observed loss of nine out of ten silent mutations originally present in the cell culture-derived virus stock used for inoculation may reflect host-specific selective pressures acting on the viral genome. These mutations could have emerged as adaptations during virus passage in human SW13 cell culture, potentially enhancing replication efficiency in vitro. Their reversions in the virus isolated from the animal host may indicate that these mutational changes confer no advantage for replication in vivo. At the same time, since virus re-isolation was performed in human SW13 cells, rather than using sheep-derived cell culture systems or direct sequencing from sheep-derived material, an influence of cell culture-associated selection on the observed sequence changes cannot be excluded. Although the observed mutations are synonymous and do not alter the encoded amino acid sequence, previous studies suggest that synonymous mutations can, in some cases, influence viral fitness by influencing RNA secondary structure, codon usage bias, or interactions with host cellular factors, thereby potentially impacting processes such as RNA stability and translation efficiency. A recent study has highlighted the role of mRNA structure in regulating viral replication dynamics^51^, supporting the idea that even synonymous changes can have functional consequences. However, no functional relevance has so far been described for the specific genome positions identified in this study. The differential mutational selection in cell culture environments versus natural hosts emphasizes the complexity of viral adaptation and underlines the need for careful interpretation of mutations observed in vitro. Further investigations are warranted to elucidate whether these silent mutations contribute to viral replication, host adaptation, or pathogenesis in natural hosts.

Taken together, the presence of infectious virus particles in the blood of the sheep experimentally infected with CCHFV suggests that small ruminants naturally infected with CCHFV through tick bites may represent a potential source of human infection, e.g., during slaughter, necropsy, birthing or similar events. While no vaccines for use in humans or livestock are currently approved, several candidates are under development, and it remains to be determined whether any of them can effectively reduce or block viremia in sheep and thereby help prevent infection and transmission. The greater understanding of CCHFV infection dynamics in sheep provided by this study will contribute to the development and evaluation of effective countermeasures such as vaccines and antivirals. Moreover, our findings highlight the need for further studies to elucidate the underlying mechanisms that allow efficient control of virus replication and enable us to determine correlates of protection. The study also helps address a substantial knowledge gap that still exists regarding the pathogenicity of CCHFV in one of its natural host species, the sheep.

## Methods

### Biosafety and animal ethics

All infectious work with CCHFV was performed in the biosafety level 4 (BSL4) laboratory at the Friedrich-Loeffler-Institut. All animal work was approved by the competent national authority of the Federal State of Mecklenburg-Western Pomerania, Germany (file number: 7221.3-1-035/23), and performed in strict accordance with national (Tierschutzgesetz, Tierschutz-Versuchstier-Verordnung) and European (RL 2010/63/EU) legislation.

### Cells and virus stocks

Human adrenocortical carcinoma (SW13) cells were maintained in Leibovitz-15 (L-15) medium supplemented with 5% fetal calf serum (FCS; L-15–5) and incubated at 37 °C without CO_2_. The CCHFV isolate Kosovo Hoti was kindly provided by Dr. Heinz Feldmann (Rocky Mountain Laboratory, Montana) and passaged once in SW13 cells to produce a virus stock for animal inoculation and virus neutralization test (VNT). Virus titers were determined by plaque assay using SW13 cells^29^. Viral nucleic acid from the challenge virus was extracted and sent to Eurofins Genomics Europe Sequencing GmbH, Germany, where deep sequencing was performed using the Illumina NovaSeq platform. Sequences were aligned to the available sequences on GenBank (GenBank accession numbers DQ133507, EU037902, EU044832). Sequence analysis revealed a total of ten synonymous mutations in the CCHFV Hoti stock as shown in Supplementary Table 3.

Human embryonic kidney 293T cells (HEK-293T, FLI, CCLV-RIE 0197) were cultivated in Minimal Essential Medium (Earl’s and Hank’s salts 1:1) supplemented with 1% non-essential amino acids, 0.125% sodium hydrogen carbonate, 0.012% sodium pyruvate and 10% FCS and incubated at 37 °C with 5% CO_2_.

### Study design

Eight domestic sheep (*Ovis aries*) of the Pomeranian Coarsewool breed, male and approximately five months old, were obtained from a local farm in Mecklenburg Western Pomerania. Water was available *ad libitum*. Hay cobs were fed once a day. Six sheep were challenged with 2 × 10^5^ TCID_50_ CCHFV Kosovo Hoti through a combined intravenous and subcutaneous inoculation. The challenge dose was confirmed by back-titration of the inoculum on SW13 cells. Two sheep (SM 1, SM 2) were mock-infected using cell culture medium and the same inoculation route. A daily physical examination of all sheep included clinical scoring and measuring the body temperature. Clinical scoring comprised assessment of physical condition, including respiration, excretion, bleeding, injuries, and body temperature; neurological signs, including limb movement; and behavior, including activity, body posture, food intake, reflexes, and response to physical contact. In addition, the health status of all animals was monitored by video at least once daily. At predetermined days (Supplementary Fig. 1), blood and swab samples were collected. Prior to infection, sheep were assigned to predefined early and late necropsy groups. For euthanasia, sheep were first sedated with xylazine (0.05 mg/kg body weight, intramuscularly; CP-Pharma) and subsequently euthanized by intravenous administration of pentobarbital (500 mg/kg body weight; WDT (Wirtschaftsgenossenschaft deutscher Tierärzte eG). One sheep was necropsied at day five and two on day seven post-infection (dpi; Group Early: SEI 1-3), while three sheep were necropsied at 20 and 21 dpi (Group Late: SLI 1-3). The two mock-infected sheep were necropsied at 7 (SM 1) and 21 dpi (SM 2). During necropsies, organs were collected for virologic and pathologic analyses. For data presentation, the tissue panel was grouped in the following tissue regions: Tissue Region I: mandibular lymph node, salivary gland, trachea, tonsil, thyroid gland, esophagus, brain, nose, eye; Tissue Region II: heart I, heart II, lung: left cranial lobe, right cranial lobe, bronchus left, bronchus right, tracheobronchial lymph node, thymus, superficial cervical lymph node, spleen, liver with gall bladder, liver; Tissue Region III: reticulum, rumen, omasum, abomasum, duodenum, pancreas, jejunum, mesenteric lymph node, ileum, caecum, colon, adrenal gland, kidney, urinary bladder, testicle, nervus ischiadicus.

### Digital droplet PCR for quantification of copy numbers and for generating a PCR standard

Digital droplet PCR (ddPCR) was used to accurately determine the number of CCHFV viral genome copies present in a viral RNA isolate, in order to create a standard suitable for high-throughput testing. ddPCR was performed using a QX200 ddPCR system (Bio-Rad Laboratories, Inc.) with a One-Step RT-ddPCR Advanced Kit for Probes (Bio-Rad Laboratories, Inc.), following the manufacturer’s instructions. In brief, 5 µl of Supermix were mixed with 2 µl of reverse transcriptase (20 U/µl) and 1 µl of 300 mM DTT. Then, a primer-probe working solution (final concentrations: 900 nM primer and 250 nM probe; primer and probe same as used in the section “CCHFV RNA detection”) was added, bringing the volume up to 15 µl, and 5 µl of the CCHFV Kosovo Hoti strain RNA isolate were added. This mixture was added to the cartridge for droplet generation, to which 70 µl of Droplet Generation Oil for Probes was added into the corresponding wells. Droplets were generated according to the manufacturer’s instructions, after which the suspension was transferred to a PCR plate. The PCR conditions consisted of 1 h for reverse transcription and 10 min for enzyme activation, followed by 40 cycles of 30 s at 95 °C and 1 min at 55 °C. Finally, the enzymes were deactivated for 10 min at 98 °C, after which the reaction mixture was cooled to 4 °C. The droplets were then analyzed using the Qx200 Reader and evaluated using the Qx Manager Standard Edition software. The obtained values were then used to create dilutions for a standard dilution series for high-throughput RT-qPCR analysis of RNA isolates. Analysis of the standard dilution series indicated a detection limit of approximately 1 RNA copy per µl of extracted RNA.

### CCHFV RNA detection

Total RNA was extracted from serum and swab samples using QIAmp Viral RNA extraction kits (Qiagen, Hilden, Germany) according to manufacturer’s instructions. For RNA extraction from sodium citrate-treated whole blood, blood samples were diluted in PBS (1:1) before manufacturer’s protocol was followed. Tissue samples were homogenized and inactivated in Trizol LS (Thermo Fisher Scientific Inc.) by a TissueLyser (Qiagen) before removal from BSL4 laboratory.

For RNA extraction from tissue homogenates, 200 µl chloroform were added to the sample, mixed well, and then incubated for 10 min at RT before centrifugation at 7000 rpm for 10 min at 4 °C. The aqueous phase was mixed with an RNA carrier and 750 µl isopropanol before incubation for 10 min at RT followed by centrifugation at 13,000 rpm for 10 min at 4 °C. The pellet was mixed with 500 µl 70% ethanol and centrifuged at 7500 rpm for 5 min at RT. The pellet was dried for 15 min at RT before resuspension in 50 µl RNase free water.

The presence of CCHFV RNA was determined by quantitative real-time one-step RT-PCR (RT-qPCR) using primers and probe as described in Atkinson et al.^52^. Using the AgPath-IDTM One Step RT-PCR kit (Thermo Fisher Scientific Inc.), the final master mix comprised 7.5 µl of 2x RT-PCR buffer, 2.5 µl of a primer-probe working solution (10 µM primer/0.3125 µM probe working solution), 1.4 µl of PCR-grade water, and 0.6 µl of 25x RT-PCR enzyme mix. Then, 3 µl of template RNA was added to give a final volume of 12 µl. Cycling conditions were as follows: 48 °C for 10 min, 95 °C for 10 min, followed by 45 cycles of 95 °C for 15 s and 52.8 °C for 30 s with the quantification analysis of fluorescence performed at the end of each 52.8 °C step.

### Virus titration and isolation

Virus titration from CCHFV PCR positive serum samples was performed using SW13 cells with 80% confluency in 96-well plates. Medium was removed from cells and replaced by 100 µl L-15 with 2% FCS and penicillin/streptomycin. In addition, ten-fold dilutions of the respective serum in a total volume of 100 µl per well were added. The plate was incubated at 37 °C. All cells were read for cytopathic effects after an incubation time of 6 days.

For virus titration from CCHFV PCR positive tissue samples, tissues were homogenized in 1 ml L-15 with a 5 mm stainless steel bead at 30 Hz for 2 min. After centrifugation at 20,000 × *g* for 5 min, ten-fold dilutions of homogenates were used to infect SW13 cells in 96-well plates as above. All cells were read for cytopathic effects at day 6. PCR-negative samples from mock-infected animals served as controls and were used to assess cytotoxicity of the respective samples.

Additionally, virus was isolated from the serum sample of sheep SLI 3, collected at 3 dpi and displaying the highest viral load as determined by PCR, for sequence analysis to assess potential mutations in the viral genome by comparing it to the inoculum. The serum was diluted 1:100 in L-15 medium with 2% FCS and penicillin/streptomycin and applied to SW13 cells seeded in a 6-well plate. The cells were incubated with the diluted serum for 1 h at 37 °C. After incubation, the inoculum was removed, and the cells were washed three times with PBS. Fresh L-15 medium supplemented with 2% FCS and penicillin/streptomycin was then added, and the cells were incubated at 37 °C for 4 days. Following incubation, the supernatants were collected, and viral RNA was extracted using the QIAamp Viral RNA Kit (Qiagen). The RNA was subsequently sent to Eurofins Genomics Europe Sequencing GmbH, Germany, for deep sequencing as described above. Sequences were aligned to the available sequences on GenBank (GenBank accession numbers DQ133507, EU037902, EU044832).

### Blood chemistry

Blood chemistry was analyzed on a VetScan VS2® (Zoetis Inc., Pasippany, New Jersey, United States) using the Preventive Care Profile Plus rotor with serum according to manufacturer’s instructions. Analyses included liver and kidney markers.

### Histopathology and immunohistochemistry

Tissue samples from all animals were collected during necropsy. Sampling sites included brain, heart, lung (including adjacent lymph node), kidney, liver, esophagus (7/8 animals), reticulum, rumen, omasum, abomasum, small and large intestine (including mesenterial lymph nodes), skin and muscle tissue at the site of infection (7/8 animals), spleen, adrenal gland and trachea (6/8 animals). Samples were fixed in 4% formaldehyde for at least 21 days with one additional change of formaldehyde, embedded in paraffin wax (FFPE tissue), and cut to 2 µm thick slides. Hematoxylin and eosin (HE) staining was performed following routine protocols^53^.

Immunohistochemistry (IHC) analysis was performed on lung, liver, testicle, spleen, and kidney samples from all animals, utilizing antibodies against CCHFV N (3A7B2) or CCHFV Gc (5B5C1A; both FLI Biobank). In short, epitope retrieval was reached by microwave heating for 20 min in citrate buffer (pH: 6.00), followed by a blocking step with goat serum. The primary antibody was diluted 1:400, incubated on the slides for 1 h at RT and marked with a biotinylated anti mouse antibody (Vector Laboratories, Newark, USA) diluted 1:200 and applied for 30 min. Staining was done by using the Vectastain Elite ABC Kit (Vector Laboratories) according to the manufacturer’s instructions and following application of AEC (Abcam, Cambridge, UK) as a chromogen. Hematoxylin was used for counterstaining. CCHFV-positive and negative cell pellets served as positive or negative controls, respectively (see Fig. 4).

Evaluation of slides was done with a digital slide scanning system (Hamamatsu Deutschland, Herrsching, Germany) and ndp.view2 software (version 2.9.29; Hamamatsu photonics, Herrsching, Germany). Alterations and IHC signals were scored according to a semi-quantitative degree with 4 tiers: 0: no alterations, 1: minimal (<5% of the area affected or 1–3 foci), 2: mild (6–40% or >3 foci), 3: moderate (41–80%, or coalescing), 4: severe (>80%, or diffuse). The histopathology and IHC scoring were blinded. Figure 4 was created using GIMP (GNU Image Manipulation Program, Kimball et al., Version 3.0.4).

### Antibody analyses by ELISA

Anti-N antibody levels were measured using the ID Screen^®^ CCHF Double Antigen Multi-species ELISA (IDvet; Innovative Diagnostics, Grabels, France) as well as the Panadea CCHFV (NP) IgG ELISA Kit (Panadea Diagnostics GmbH, Hamburg, Germany), following the manufacturers’ protocols.

For the in-house IgM ELISA, Nunc Polysorp 96-well plates were coated overnight at 4 °C with rabbit anti-sheep IgM antibody (Bio-Rad), diluted 1:100 in 0.05 M carbonate-bicarbonate buffer (pH 9.6). Empty mock wells coated only with 0.05 M carbonate-bicarbonate buffer served as control wells to evaluate unspecific binding of the sera. After coating, plates were blocked with 10% skim milk in PBS for 1 h at 37 °C, followed by three washes using PBS with 0.1%Tween20. Serum samples were diluted 1:5 in 2% skim milk and added in duplicate to both antibody-coated and control wells, and incubated for 1 h at 37 °C. After another washing step, HRP-labeled CCHFV-NP from the Panadea CCHFV (NP) IgG ELISA Kit (as described in ref. ^31^) was diluted 1:1400 in 2% skim milk and added to the plates for 1 h at 37 °C. After a final washing step, plates were incubated with 1-Step™ Ultra TMB (Thermo Fisher Scientific Inc.) for 20 min at RT and stopped with equal amounts of 1 M sulfuric acid. The readout was performed as described for the in-house IgG ELISAs.

For in-house IgG ELISAs, Greiner F medium binding 96-well plates were coated with either commercial recombinant Gn or Gc (both derived from CCHFV IbAr 10200, The Native Antigen Company, Kidlington, UK), or with in-house produced CCHFV mucin-like domain (MLD)-GP38-Strep^®^. Recombinant CCHFV MLD-GP38-Strep^®^ (in the following referred to as CCHFV GP38) was transiently expressed in HEK 293T cells and purified from cell culture supernatant by Strep-tag affinity chromatography. Briefly, a gene fragment from CCHFV glycoprotein precursor (GPC, Kosovo Hoti) encoding aa 1-519 (_1_MLD-GP38_519_) was fused to a C-terminal Twin-strep-tag, codon-optimized and synthesized (Thermo Fisher Scientific, Waltham, USA) for expression in human cells before subcloning into the expression vector pcDNA 3.1. HEK 293T cells were transfected using polyethylenimine (PEI) in a DNA-PEI ratio of 1:3. Supernatants were harvested at 72 h post transfection, and purified using the Twin-Strep-tag and Strep-Tactin XT 4Flow-based affinity chromatography according to manufacturer’s instructions.

For coating, antigens were diluted (CCHFV GP38 200 ng/Well; Gn and Gc 100 ng/Well) in 0.01 M PBS. To evaluate unspecific binding of the sera, supernatants from mock transfected HEK 293T cells were purified as described above and used in equal amount as mock antigen. After blocking the wells with ID Vet blocking buffer for 1 h at 37 °C, each serum sample was diluted 1:100 in IDvet Dilution Buffer No. 14 and added in duplicate to both the control and antigen containing wells. After incubation for 1 h at 37 °C followed by three washes using PBS with 0.05% Tween20 (PBST), Protein G conjugate labeled with horseradish peroxidase (HRP; Merck Millipore, Billerica, USA) diluted 1:5000 in IDvet Dilution Buffer No.3 was added to incubate for another 1 h at 37 °C. A rabbit anti sheep HRP (Southern Biotechnology Associates Inc, Birmingham, USA) antibody was used in a 1:40,000 dilution as conjugate for the Gc ELISA. After another three washes and an incubation with 3,3’,5,5’-Tetramethylbenzidine (TMB) peroxidase substrate (Bio-Rad; Bio-Rad Laboratories, Inc., Hercules, USA) for 10 min, the reaction was stopped with equal amounts of 1 M sulfuric acid. Absorbance was measured at 450 nm. For all samples, the corrected OD_450 nm_ value was calculated as (mean OD_450 nm_ value with antigen) – (mean OD_450 nm_ value without antigen).

### Virus neutralization test (VNT)

The neutralizing activity of the serum was determined by VNT as described before^29^. Briefly, heat-inactivated serum samples were serially diluted in L-15 medium starting from 1:2. Serial dilutions were mixed with approximately 150 plaque forming units (pfu) of CCHFV Kosovo Hoti per well and incubated for 1 h at 37 °C. After that, the virus-serum mix was added to SW13 cells followed by another incubation at 37 °C for 6 days. Neutralizing activity was assumed in wells where a cytopathic effect was completely neutralized. The neutralizing titer of a serum was calculated as arithmetic mean titer of three replicates that showed neutralizing activity.

### Interferon gamma (IFN-γ) ELISpot assay

IFNγ ELISpot assays using bovine IFN‑γ ELISpot PLUS kits (Mabtech, Nacka Strand, Sweden) were performed on peripheral blood mononuclear cells (PBMCs) isolated via Pancoll gradient centrifugation with Sheep SM 2 and SLI 1-3.

Briefly, 25 ml heparinized blood was diluted 1:1 with PBS. In the next step, the diluted blood was layered on Pancoll (PAN-Biotech GmbH, Aidenbach, Germany). After centrifugation, the PBMCs were removed from the interphase and washed once with PBS. After a further centrifugation step, residual erythrocytes were lysed with ACK buffer (Thermo Fisher Scientific Inc.) according to the manufacturer’s instructions. After a final washing step with PBS, the PBMCs were split into 3 aliquots and frozen in liquid nitrogen until further analysis. For IFNγ ELISpot assays, PBMCs were resuspended in 5 ml of X-Vivo 15 medium with penicillin / streptomycin (X-Vivo 15 P/S) and incubated for 1 h at room temperature (RT). In the meantime, the commercial ELISpot 96-well plate was washed four times with PBS and incubated afterwards for at least 30 min with X-Vivo 15 P/S. After that, medium was removed and 100,000 cells were added per well. Next, 15-mer peptides overlapping by 11 amino acids spanning either the entire GPC (Peptides & Elephants, Hennigsdorf, Germany) or the N (GenScript Biotech, Piscataway Township, USA) were pooled into groups of 22–34 peptides per pool and added to the respective wells. Cells were stimulated with peptide pools at a final concentration of 1 μg/mL per peptide. As positive controls, cells were stimulated with concanavalin A (ConA) or Phytohemagglutinin-L (PHA-L) (1:500; Thermo Fisher Scientific Inc.). Negative control wells received an equivalent volume of medium alone. Cells were incubated for 36 h at 37 °C in 5% CO_2_. After incubation, the following steps were carried out according to the manufacturer´s instruction. Briefly, cell supernatants were removed and plates were washed with PBS. After that, plates were incubated with an IFNγ-specific biotin-labeled antibody (MT307-biotin; Mabtech) in a concentration of 2.5 µg/well and incubated for 2 h at RT. After a next washing step, plates were incubated with 100 µl Streptavidin-ALP (1:1000, Mabtech) per well and incubated for another 1 h at RT. After a final washing step, plates were incubated with the substrate solution BCIP/NBT-plus, incubating 100 µl for 10 min at RT. After that, plates were washed extensively in tap water and dried before readout. Developed spots indicating IFNγ production were counted using an AID ELISpot Reader (AID Autoimmun Diagnostika GmbH, Straßberg, Germany). Spot counts were normalized to 1 × 10^6^ PBMCs. Background signal, defined as spot counts in medium only wells, was subtracted from peptide- or control-stimulated wells to result in corrected spot forming units (SFU). All measurements were performed in duplicate. GPC pools covered the following antigens: MLD: GPC 1–3; GP38: GPC 3–5; Gn: GPC 5–7; NSm: GPC 7–9; Gc: 9–14.

## Supplementary information

Supplementary information

## Data Availability

The dataset generated during the current study have been deposited in a Zenodo repository (DOI: 10.5281/zenodo.20818431) and will be made publicly available upon publication of this article. During peer review, the data are available from the corresponding author on reasonable request.
