# Supplementary material for: Viral dissemination and diagnostic insights from experimental infection of sheep with Crimean-Congo hemorrhagic fever virus
Source: Npj Viruses. 2026 Aug 24;4:39. doi: 10.1038/s44298-026-00228-y (PMC13503663; doi:10.1038/s44298-026-00228-y)
Supplement: Supplementary file 1 [file 44298_2026_228_MOESM1_ESM.pdf]

Supplements

Supplementary Figure 1: Schematic overview of the study design. Sheep were inoculated with CCHFV (Kosovo Hoti) on day 0. Blood samples and swabs were collected at the indicated time points before and after infection (days post infection, dpi). Necropsies were performed at designated dpi.

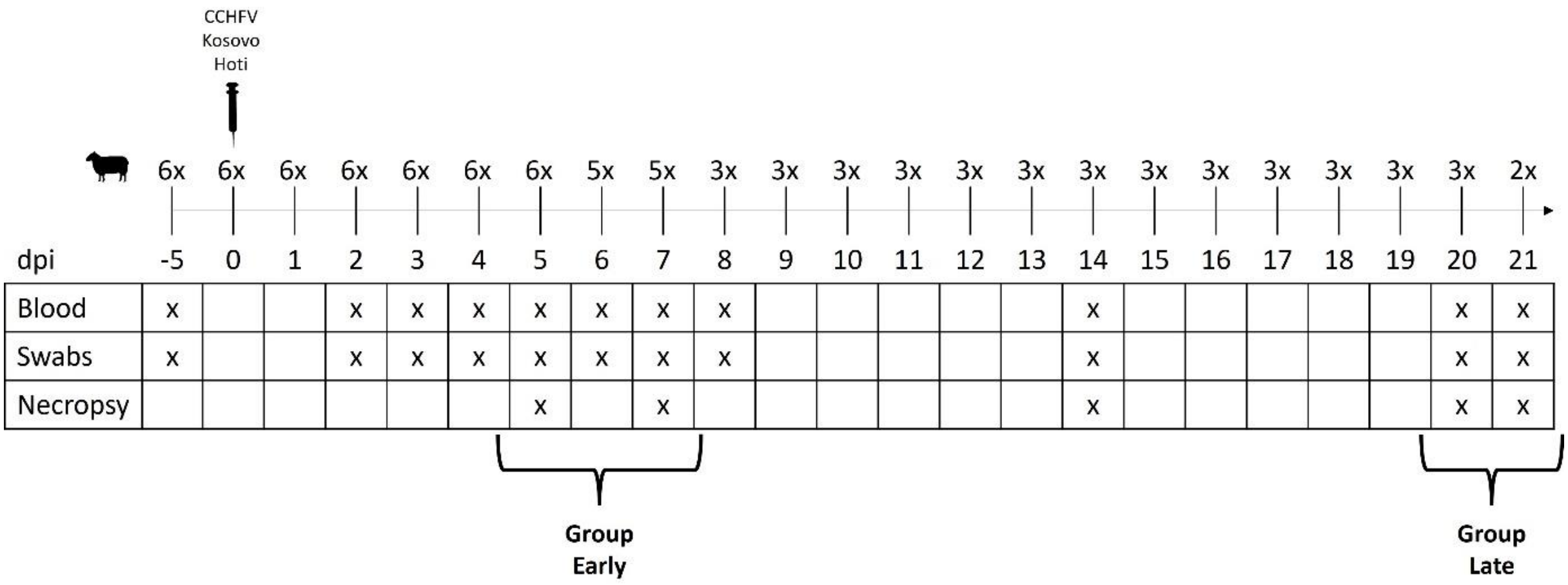

Supplementary Figure 2: CCHFV RNA loads determined by RT-qPCR in nasal (A), oral (B) and rectal (C) swabs. Copy numbers are expressed as $\log_{10}$ copies per milliliter (ml) of swab buffer.

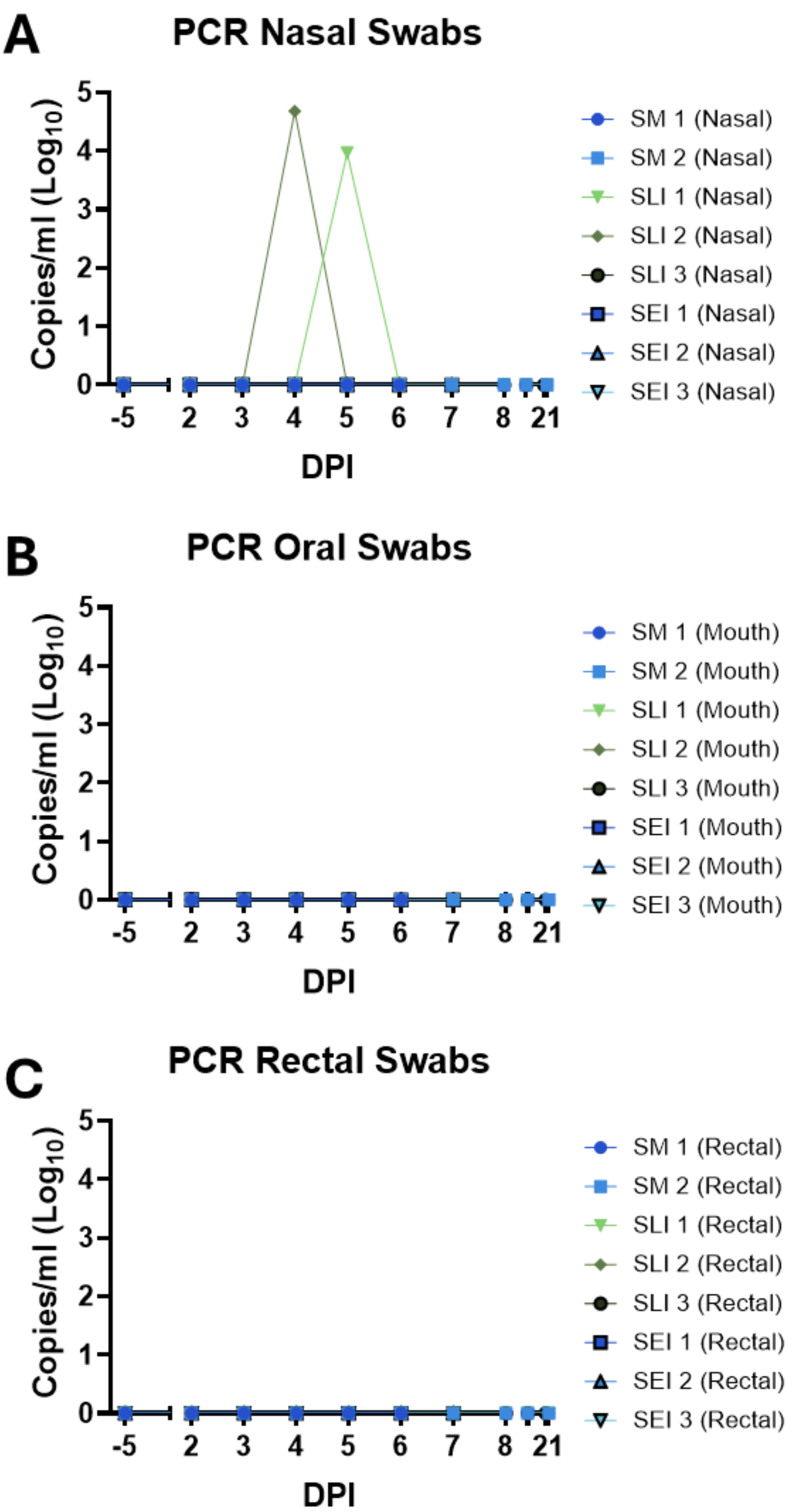

Supplementary Table 1: Clinical scores of the affected categories of mock (A) and CCHFV (B)-infected sheep during the period in which clinical signs were observed, despite of rectal body temperature. All animals had clinical scores of 0 for the remainder of the study.
Score 0 = physiological, 1 = mild symptom, 2 = moderate symptom. 3 = severe symptom (humane endpoint)

**A**		**Score**	**-1 dpi**	**0 dpi**	**1 dpi**	**2 dpi**	**3 dpi**	**4 dpi**	**5 dpi**	**6 dpi**	**7 dpi**
Respiration	Physiological	0	2/2	2/2	2/2	2/2	2/2	2/2	2/2	2/2	2/2
	Mildly increased	1	0/2	0/2	0/2	0/2	0/2	0/2	0/2	0/2	0/2
	Moderately increased	2	0/2	0/2	0/2	0/2	0/2	0/2	0/2	0/2	0/2
	Severely increased	3	0/2	0/2	0/2	0/2	0/2	0/2	0/2	0/2	0/2
Food intake	Eagerly eats the offered food	0	2/2	2/2	2/2	2/2	2/2	2/2	2/2	2/2	2/2
	Eats the offered food hesitantly	1	0/2	0/2	0/2	0/2	0/2	0/2	0/2	0/2	0/2
	Does not eat the offered food but sniffs at it	2	0/2	0/2	0/2	0/2	0/2	0/2	0/2	0/2	0/2
	Does not eat; shows no interest in food	3	0/2	0/2	0/2	0/2	0/2	0/2	0/2	0/2	0/2
Excretion	Well-formed	0	0/2	0/2	0/2	0/2	0/2	0/2	0/2	0/2	0/2
	Loose	1	2/2	2/2	2/2	2/2	2/2	2/2	2/2	2/2	2/2
	Diarrhea	2	0/2	0/2	0/2	0/2	0/2	0/2	0/2	0/2	0/2
	Watery, bloody diarrhea (>24 h)	3	0/2	0/2	0/2	0/2	0/2	0/2	0/2	0/2	0/2

B

		Score	-1 dpi	0 dpi	1 dpi	2 dpi	3 dpi	4 dpi	5 dpi	6 dpi	7 dpi
Respiration	Physiological	0	0/6	0/6	4/6	1/6	4/6	4/6	1/6	2/5	5/5
	Mildly increased	1	0/6	0/6	2/6	5/6	2/6	2/6	5/6	3/5	0/5
	Moderately increased	2	0/6	0/6	0/6	0/6	0/6	0/6	0/6	0/5	0/5
	Severely increased	3	0/6	0/6	0/6	0/6	0/6	0/6	0/6	0/5	0/5
Food intake	Eagerly eats the offered food	0	6/6	6/6	6/6	0/6	0/6	0/6	0/6	5/5	5/5
	Eats the offered food hesitantly	1	0/6	0/6	0/6	6/6	6/6	6/6	6/6	0/5	0/5
	Does not eat the offered food but sniffs at it	2	0/6	0/6	0/6	0/6	0/6	0/6	0/6	0/5	0/5
	Does not eat; shows no interest in food	3	0/6	0/6	0/6	0/6	0/6	0/6	0/6	0/5	0/5
Excretion	Well-formed	0	5/6	6/6	6/6	4/6	5/6	4/6	4/6	4/5	5/5
	Loose	1	1/6	0/6	0/6	2/6	1/6	2/6	2/6	1/5	0/5
	Diarrhea	2	0/6	0/6	0/6	0/6	0/6	0/6	0/6	0/5	0/5
	Watery, bloody diarrhea (>24 h)	3	0/6	0/6	0/6	0/6	0/6	0/6	0/6	0/5	0/5

Supplementary Table 2: Overview of the virus titration results. RT-qPCR-positive serum samples were subjected to virus titration. Titers are expressed as $TCID_{50}$/ml, with corresponding RT-qPCR values shown in brackets as RNA copies/ml. n.t., not tested.

	SEI 1	SEI 2	SEI 3	SLI 1	SLI 2	SLI 3
	$TCID_{50}$/ml (Copies/ml)	$TCID_{50}$/ml (Copies/ml)	$TCID_{50}$/ml (Copies/ml)	$TCID_{50}$/ml (Copies/ml)	$TCID_{50}$/ml (Copies/ml)	$TCID_{50}$/ml (Copies/ml)
-5 DPI	n.t. (0)	n.t. (0)	n.t. (0)	n.t. (0)	n.t. (0)	n.t. (0)
2 DPI	562.34 (1467.67)	5623.41 (16413.33)	1000.00 (5373.33)	5623.41 (7786.67)	1778.28 (6896.67)	10000.00 (6283.33)
3 DPI	0.00 (1435.33)	56.23 (2289.33)	n.t. (0)	562.34 (10276.67)	562.34 (12203.33)	1000.00 (127566.67)
4 DPI	1778.28 (25103.33)	3162.28 (63400.00)	n.t. (0)	17782.79 (27013.33)	316.23 (11516.67)	1778.28 (12996.67)
5 DPI	n.t. (0)	177.83 (824.33)	n.t. (0)	n.t. (0)	n.t. (0)	56.23 (4023.33)
6 DPI	0.00 (2039.00)	n.t. (0)		n.t. (0)	n.t. (0)	0.00 (2056.33)
7 DPI	n.t. (0)	n.t. (0)		0.00 (105.00)	0.00 (625.00)	0.00 (136.57)
8 DPI				n.t. (0)	n.t. (0)	n.t. (0)
14 DPI				n.t. (0)	n.t. (0)	n.t. (0)
20/21 DPI				n.t. (0)	n.t. (0)	n.t. (0)

Supplementary Table 3: Overview of the ten synonymous mutations in the virus stock used for inoculation: This table lists mutations identified in the viral inoculum, categorized by the S, M, and L segments of the CCHFV genome. Mutation positions correspond to nucleotide coordinates based on reference sequences (GenBank accession numbers DQ133507, EU037902, EU044832).

SAMPLE	Ref	POSITION	REFERENCE BASE	OBSERVED BASE
CCHFV_Kosovo_Hoti	DQ133507.1 (S)	436	A	G
CCHFV_Kosovo_Hoti	EU037902.1 (M)	763	T	C
CCHFV_Kosovo_Hoti	EU037902.1 (M)	2799	G	A
CCHFV_Kosovo_Hoti	EU037902.1 (M)	2827	A	G
CCHFV_Kosovo_Hoti	EU044832.1 (L)	3148	A	G
CCHFV_Kosovo_Hoti	EU044832.1 (L)	8135	C	T
CCHFV_Kosovo_Hoti	EU044832.1 (L)	9919	C	T
CCHFV_Kosovo_Hoti	EU044832.1 (L)	10828	G	A
CCHFV_Kosovo_Hoti	EU044832.1 (L)	11238	A	G
CCHFV_Kosovo_Hoti	EU044832.1 (L)	11618	G	A

Supplementary Table 4: Overview of the three synonymous mutations detected in the viral isolate obtained from serum of SLI 3 at 3 dpi: This table lists mutations identified in the viral isolate, categorized by the S, M, and L segments of the CCHFV genome. Mutation positions correspond to nucleotide coordinates based on reference sequences (GenBank accession numbers DQ133507, EU037902, EU044832).

SAMPLE	Ref	POSITION	REFERENCE BASE	OBSERVED BASE
SLI 3	EU037902.1 (M)	3445	G	A
SLI 3	EU044832.1 (L)	2964	C	T
SLI 3	EU044832.1 (L)	3148	A	G

Supplementary Table 5: Serum blood chemistry from one mock (SM 2) and three CCHFV-infected sheep (SLI 1-3). Serum samples were collected at the indicated days post infection (dpi) and analyzed on a VetScan VS2® (Zoetis Inc., Pasippany, New Jersey, United States) using the Preventive Care Profile Plus rotor according to manufacturer's instructions. The green color gradient highlights relative changes in serum chemistry values, with darker shades indicating higher concentrations. BUN = blood urea nitrogen; CRE = creatinine; ALT = alanine aminotransferase; ALP = alkaline phosphatase; AST = aspartate aminotransferase; TBIL = total bilirubin; GLU = glucose; CA = calcium; TP = total protein; ALB = albumin; GLOB = globulin; Na+ = sodium; K+ = potassium; Cl- = chloride; tCO_2 = total carbon dioxide.

Animal ID	DPI	BUN mmol/L	CRE µmol/L	ALT U/L	ALP U/L	AST U/L	TBIL µmol/L	GLU mmol/L	CA mmol/L	TP g/L	ALB g/L	GLOB g/L	Na+ mmol/L	K+ mmol/L	Cl- mmol/L	tCO2 mmol/L
Reference		4.5 – 10.7	50 – 120	< 33	< 359	< 126	< 8.5	2.2 – 5.2	2.1 – 2.7	50 – 70	24 – 30	< 48	145 – 155	3.5 – 4.5	75 – 114	-
SM 2	-5	5	80	15	331	139	5	5	3.18	81	39	41	158	5.8	104	35
SM 2	2	3.2	83	25	302	155	5	5	3.41	85	41	44	161	6.1	109	36
SM 2	3	2.6	64	18	261	123	4	4.3	2.85	68	33	35	150	5.3	111	33
SM 2	4	3.2	67	17	249	132	5	4.9	3.12	75	36	38	152	5.9	103	33

SM 2	5	3.5	90	16	210	114	5	4.5	3.06	71	34	37	159	6.1	114	34
SM 2	7	3.1	48	17	277	115	5	4.3	2.93	71	34	37	142	5.8	102	32
SM 2	8	2.9	77	17	266	121	3	4.8	3.09	74	35	38	150	6.1	109	34
SM 2	14	2.9	65	15	163	95	4	4.2	2.69	61	28	34	143	6.1	104	29
SM 2	21	2.8	84	20	202	132	5	5.3	2.99	74	33	41	154	5.7	104	33
SLI 1	-5	3.8	75	12	212	106	4	3.9	3.16	67	32	35	152	6.3	98	35
SLI 1	2	2.6	74	10	200	91	4	4.2	2.5	57	21	36	146	5.1	103	32
SLI 1	3	6.1	69	6	164	93	4	4.2	2.52	59	21	38	149	5.4	107	32
SLI 1	4	3.2	68	6	172	92	4	4.5	2.6	61	22	39	154	5.7	108	32
SLI 1	5	6.2	75	13	207	87	5	4.1	2.48	57	20	38	146	5.1	101	32
SLI 1	7	2.4	58	8	157	84	4	3.8	2.39	50	17	33	134	4.4	92	31

SLI 1	8	5.8	70	7	188	105	4	4	2.82	60	21	39	163	6	112	34
SLI 1	14	4	56	11	178	71	4	3.6	2.49	54	18	36	140	5.5	99	31
SLI 1	21	2.2	52	11	152	75	3	5.3	2.78	60	24	36	144	5.9	101	31
SLI 2	-5	2.4	78	13	309	87	4	3.7	3.02	66	34	32	150	6	98	33
SLI 2	2	2.4	57	13	233	76	4	4.4	2.57	64	29	36	147	5.3	106	32
SLI 2	3	4.7	56	7	268	83	4	4.3	2.73	72	32	40	163	6.2	114	34
SLI 2	4	2.5	61	<5	187	71	4	4.1	2.46	59	25	34	134	4.4	100	31
SLI 2	5	3.9	67	11	175	70	4	3.8	2.4	59	24	35	136	4.5	98	30
SLI 2	7	2.4	38	<5	145	84	4	3.5	2.41	58	23	34	132	4.5	94	29
SLI 2	8	4.4	49	10	175	105	4	4.1	3.01	73	30	43	170	6.4	119	35
SLI 2	14	2.2	39	5	194	56	4	2.8	2.31	53	20	33	118	4.4	82	29

SLI 2	21	2.4	61	11	306	87	3	4.7	3.03	65	30	35	155	5.6	108	32
SLI 3	-5	3.1	80	14	345	101	4	3.8	2.78	67	33	34	138	5.1	99	31
SLI 3	2	5	70	8	175	82	4	3.9	2.58	54	24	30	146	5.3	110	31
SLI 3	3	7.1	59	12	209	87	4	3.8	2.64	55	24	31	147	5.4	110	32
SLI 3	4	2.9	88	11	257	102	4	4.7	2.75	59	27	33	158	5.9	110	34
SLI 3	5	4.7	58	9	164	92	4	4.3	2.81	55	24	30	154	6	108	34
SLI 3	7	2.9	78	8	125	92	4	4.3	2.9	56	24	32	149	6.1	103	32
SLI 3	8	7.4	73	8	161	119	5	4.8	3.55	74	32	42	180	7.6	129	37
SLI 3	14	3.8	43	13	143	70	4	2.8	2.44	48	19	29	115	4.7	80	27
SLI 3	20	2.7	52	12	173	69	3	3.5	2.31	49	21	28	127	4.3	87	33

Supplementary Table 6: Pathology findings. Organs with no alterations are not shown. Alterations and IHC signals were scored according to a semi-quantitative degree with 4 tiers: 0: no alterations, 1: minimal (<5% of the area affected or 1-3 foci), 2: mild (6%–40% or >3 foci), 3: moderate (41%–80%, or coalescing), 4: severe (>80%, or diffuse). CCHFV IHC Immunohistochemistry against Crimean-Congo hemorrhagic fever virus antigen (N and Gc); n.a.: sample not available.

Sheep	*SM 1*	*SEI 1*	*SEI 2*	*SEI 3*	*SM 2*	*SLI 1*	*SLI 2*	*SLI 3*
Group	Mock, Group early	Infection, Group early			Mock Group late	Infection, Group late		
Interstitial Pneumonia	2	1	1	1	2	1	2	0
Intestinal eosinophilia and/or tape worm infection	3	3	1	3	3	1	0	0
Myocardial sarcosporidia	0	0	0	3	0	0	0	0
Lymphohistiocytic, rarely eosinophilic infiltrations								
- *Liver*	- 1	- 0	- 1	- 1	- 0	- 0	- 0	- 1
- *Kidney*	- 0	- 0	- 0	- 0	- 1	- 1	- 0	- 0
- *Adrenal gland*	- 0	- 3	- 0	- 0	- 0	- 0	- 0	- 0
CCHFV IHC signaling								
- *Liver*	- 0	- 1	- 0	- 1	- 0	- 0	- 0	- 0
- *Testis*	- 0	- 1	- 0	- 1	- 0	- 0	- 0	- 0

- *Spleen*	- 0	- 0	- 0	- 1	- 0	- 0	- 0	- 0
- *Lung*	- 0	- 0	- 0	- 0	- 0	- 0	- 0	- 0
- *Kidney*	- 0	- 0	- 0	- 0	- 0	- 0	- 0	- 0
Inflammatory cell infiltration at the injection site	1	2	0	1	0	n.a.	3	2
